# Enhancing golf swing performance through M1-targeted transcranial direct current stimulation: a double-blind, randomized crossover study

**DOI:** 10.3389/fspor.2025.1615617

**Published:** 2025-07-16

**Authors:** Hongbin Xiang, Hwang Woon Moon, Lu Li, Kyung Yoo

**Affiliations:** ^1^ School of Physical Education, Shanghai University of Sport, Shanghai, China; ^2^ Department of Sports & Outdoors, College of Bio Convergence, Eulji University, Seongnam, Republic of Korea; ^3^ Key Laboratory of Exercise and Health Sciences of Ministry of Education, Shanghai University of Sport, Shanghai, China

**Keywords:** tDCS, motor cortex, neuromodulation, golf swing performance, motor control, long-driving distance capacity, precision

## Abstract

**Objective:**

This study investigated whether transcranial direct current stimulation (tDCS) targeting the primary motor cortex (M1) can induce acute enhancements on golf swing performance, particularly in tasks requiring long-driving distance capacity and accuracy control.

**Methods:**

Eight professional golfers participated in a double-blind, randomized, crossover trial consisting of two conditions: active tDCS (A-tDCS) and sham tDCS (S-tDCS). Stimulation was applied over the left M1 for 20 min. Participants performed 10 swings each for three tasks (driver, iron, and wedge) both pre- and post-intervention. Performance metrics included long-driving distance variables (clubhead speed, ball speed, carry distance) and accuracy-related variables (face angle, side distance, and spatial error Data were analyzed using 2 × 2 repeated-measures ANOVAs, with *post hoc t*-tests and effect sizes (Hedge's *g*) where significant interactions were found.

**Results:**

Significant Time × Condition interactions indicated that A-tDCS improved carry distance and ball speed in iron tasks and carry distance in driver task (*p* < .05), with large effect sizes (*g* > 0.8). Side deviation also decreased significantly under A-tDCS in the iron task, indicating enhanced accuracy. No stimulation-specific effects were observed for the wedge task.

**Conclusion:**

M1-targeted A-tDCS can acutely enhance golf swing long-driving distance capacity and accuracy in tasks requiring substantial force output. This technique is promising as a performance-enhancing tool for golfers, offering a low-fatigue alternative to traditional high-intensity training.

## 1 Introduction

As a sport enjoyed by more than 60 million people worldwide (1), golf uniquely blends accessibility, entertainment, and competitive engagement across all age groups, offering an integration of recreational enjoyment and competitive play. The core of this appeal is golf swing, a multi-joint movement demanding millisecond coordination to transfer kinetic energy from ground to clubhead. During an 18-hole round, swings consume ∼60% of total time (2), placing sustained mechanical strain over the players’ musculoskeletal system. Professional golfers are the typical example of this repetitive demand, performing over 1,000 swings per week to maintain their competitive level (3). This training load is correlated with injuries that may limit athletic careers such as lumbar disc degeneration and rotator cuff disorders (4, 5). Balancing technical skill development with injury prevention presents an ongoing challenge in elite-level golf and may have implications for broader athletic populations.

Current golf training programs are conducted under the guidance of existing biomechanical researches (6). Nowadays, technologies like inertial measurement unit and force plates provide quantifiable metrics of kinetics and kinematics (7, 8). Neuromuscular measurements such as electromyography are utilized to reveal the muscular activation pattern, stretch-shortening cycle, during swing process (9, 10), revealing that swing is a powerful technique made up of rapid contraction. Biomechanical methods such as 3D motion capture help us to know the sequence of force transfer, from ground to the upper extremity, and with the cooperation of X-factor (pelvis-thorax rotation) to reach the maximal momentum (11, 12). However, these approaches primarily focused on peripheral musculoskeletal components, often overlooking the role of central neural mechanisms in motor learning and performance. As a consequence, current training programs often require prolonged periods of high-volume practice to reach the skill consolidation, which is an invisible exposure to injury risks. Emerging evidence suggests the central nervous system plays a pivotal role in skill acquisition and motor control (13, 14), and the improved cerebral plasticity might offer outstanding values on enhancing motor learning efficiency while mitigating the mechanical tension from repetitions (15), which is a possible solution for the conflict between performance and injury risks.

One such approach is transcranial direct current stimulation (tDCS), a non-invasive neuromodulation technique, offering the possibility to regulate the neuroplasticity. Via mechanisms such as long-term potentiation (16) and GABA inhibition (17), tDCS gently alters brain activity and enhance cortical excitability by delivering low-intensity currents to specific areas of brain region. In sports science research, tDCS has been examined to improve the strength of simple tasks like isometric knee extension (18) and the accuracy of skillful tasks like pistol shooting (19). However, sports like golf face a different challenge that the golf swing is combination of both rapid force production and subtle accuracy control. Moreover, golfers will adjust their club selection according to strategies, making golf swing a more complicated skill. Driver swing depends on corticospinal pathways to reach optimal driving distance, while wedge shot leverages cerebellar-parietal networks for spatial accuracy (20–22). These complex challenges of golf swing make it a perfect model to examine how tDCS could improve techniques demanding both optimal force generation and precision control.

A recent systematic review and meta-analysis supports the application of anodal tDCS in sport. This comprehensive review included 19 articles covering different aspects of performance, such as strength, endurance, and visuomotor skills (23). The result revealed moderate effects in visuomotor-dominant skills, ranging from volleyball spiking, pistol shooting, to taekwondo kicking and parkour jumping. These sports share important cognitive-motor similarities with golf, including the integration of visual input, spatial judgement, and motor execution. Given these shared characteristics, tDCS may also be effective in enhancing golf performance, particularly in swing tasks that require a combination of force generation and visuomotor control.

Most existing tDCS studies chose the primary motor cortex (M1) as the stimulation area, for its role in governing voluntary force generation (24, 25). While M1 is not traditionally associated with spatial accuracy or movement planning, it contributes to force output and fatigue inhibition (26, 27), further leading to motor noise reduction, movement consistency, and fine-grained motor output (28–30). Together, these effects may indirectly support visuomotor-dominant performance, making M1 an ideal target for improving motor capacity. However, it is still unclear whether this stimulation strategy can help with sports demand both force output and fine motor control like golf, limiting how widely tDCS can be applied in sports contexts that require various motor skills.

Therefore, this exploratory study aimed to investigate whether M1-targeted tDCS can acutely enhance golf swing performance across swing types with different performance demands. Specifically, we examined: (1) whether tDCS can enhance long-driving distance capacity (e.g., clubhead speed, ball speed, carry distance); (2) whether it can improve distance and direction accuracy (e.g., face angle, directional deviation). By integrating brain stimulation with complicated motor skills, this study seeks to advance understanding of how tDCS may be applied in sports performance contexts. For elite golfers, this approach may provide them with a new way to prepare for competition. Although this study focused exclusively on elite athletes, the findings may inform future research exploring whether tDCS can reduce training volume and injury risk in broader athletic populations. Consequently, this work offers a neuroscience-based perspective on how people can improve complex motor skills while protecting their bodies, bringing together high-performance goals and long-term well-being.

## 2 Materials and methods

### 2.1 Participants

Eight professional golfers (4 males, 4 females; age: 22.3 ± 2.0 years; height: 176.6 ± 10.9 cm; body mass: 74.4 ± 15.2 kg; training years: 8.3 ± 0.7 years), all right-handed and actively competing in national-level tournaments, volunteered to participate in this study. The decision to include only professional golfers was based on the need to reduce inter-individual variability in technique, swing mechanics, and performance consistency. Elite golfers demonstrate stable and repeatable motor patterns, which is particularly important for verifying the acute and immediate effects of tDCS.

An *a priori* power analysis was conducted using G*Power 3.1 to estimate the required sample size for a 2 × 2 repeated-measures ANOVA (within-subjects) (31). To assume a medium-to-large effect size (Cohen's *f* = 0.4), an α level of 0.05, and 80% power, the analysis indicated that 12 participants would be required. However, due to the limited availability of elite-level golfers, the study was conducted with a sample of eight. Although this number falls below the recommendation, the randomized crossover design enhances statistical power by reducing intra-subject variability. Moreover, the sample size is also consistent with previous sport neuroscience studies using tDCS, which have reported significant findings with 8–12 participants in similar crossover or within-subject designs (32–34).

Health questionnaire was signed before the study to ensure all participants did not have any head trauma, neurological disorders, metallic implants, muscular dysfunction, prior brain stimulation exposure or any contraindications. After receiving a full explanation of the procedures, potential risks, and their right to withdraw, written informed consent was obtained from each participant. Ethical approval was granted by the institutional review board of Shanghai University of Sport, and all procedures met the international safety guidelines for non-invasive brain stimulation.

### 2.2 Study design

**Figure 1:**
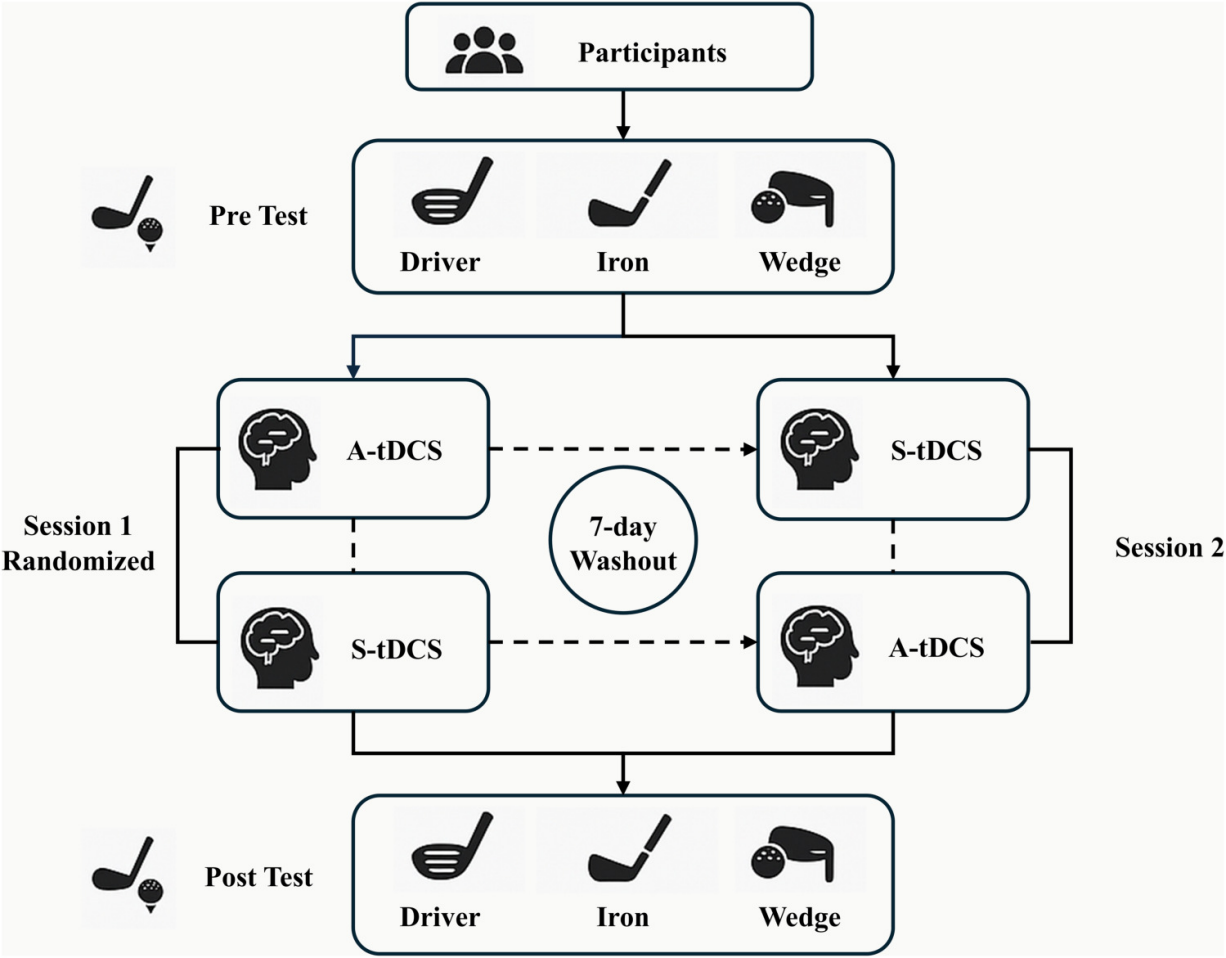
Summary of the study design.

A double-blind, randomized, counterbalanced crossover design (Figure 1) was adopted for the mitigation of individual variability and the optimization of statistical power. Two separate intervention sessions were arranged for each participant, one with active tDCS (A-tDCS) and one with sham (S-tDCS). There was a 7-day gap between two sessions, which was based on prior studies showing that tDCS effects on changing neuroplasticity typically dissipate within 3–4 days (35). By adopting a 7-day washout period, we aimed to minimize potential carryover effects and maximize the independence of each condition in this crossover design. The order of session was randomized using computer-generated blocks (block size = 4) by a researcher not involved in data collection. Throughout the study, all participants and the rest of experimenters remained blinded to condition assignment.

To minimize practice-related improvements, all participants completed a standardized warm-up and 10 familiarization swings before data collection in each session. Only performance data from the main testing block were collected. Because each participant was exposed to the same procedural structure across both conditions, any learning effects were equally distributed across the experiment. Moreover, participants were young and highly trained for long time, allowing the minimal bias caused from learning effects and fatigue. The consistent format of testing, along with the within-subject crossover design and the stable competitive level, ensured that observed performance changes were attributable to stimulation condition rather than task familiarity or fatigue accumulation.

### 2.3 tDCS protocol

The intervention session was conducted in a separate room using a DC-Stimulator Plus device (NeuroConn, GmbH, Germany) and managed by the researcher not involved in data collection to ensure the blindness. The room was monitored to maintain a stable temperature and humidity (23 ± 1°C, 50 ± 5% relative humidity).

**Figure 2:**
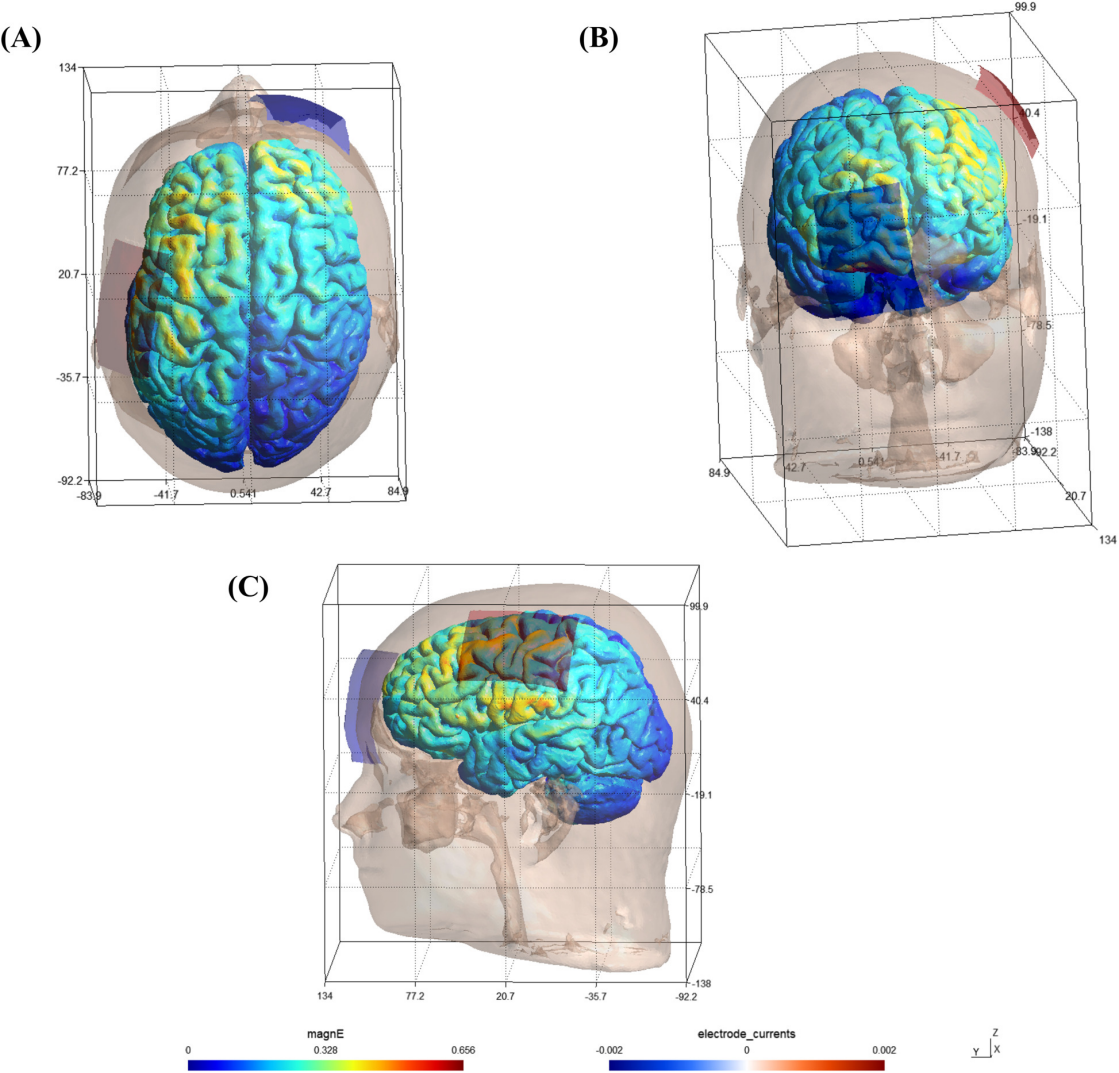
Software simulation of brain activation pattern following the targeted stimulation protocol. **(A)** Top view; **(B)** Front view; **(C)** Side view; magnE: magnitude of the electric field (Unit: V/m).

According to the international 10–20 EEG system, the anodal electrode (5 cm × 7 cm, 5 mm sponge thickness) was placed over the left M1 (EEG location: C3), while the cathodal electrode (5 cm × 7 cm, 5 mm sponge thickness) was positioned over the contralateral prefrontal cortex (EEG location: Fp2). Electrode placement was checked by anatomical landmarks to ensure the standardization across participants and simulated using SIMNIBS software (version: 4.1.0) to see the possible activation of stimulation (Figure 2).

This montage was selected based on prior research showing that anodal stimulation of the dominant hemisphere's M1 enhances excitability of corticospinal pathways, especially in right-handed individuals. Cathodal stimulation, which typically reduces cortical excitability, is less aligned with the performance enhancement objectives of this investigation. Placement of the return electrode over contralateral prefrontal cortex minimizes the likelihood of inhibitory current flow through adjacent motor areas, thus focusing stimulation on the motor cortex and avoiding bilateral motor effects (36). This configuration has been validated in several prior motor performance and sport-specific tDCS studies (23, 24).

For the A-tDCS session, a 2 mA current (density: 0.057 mA/cm^2^) was delivered to the targeted area for 20 min, with a 30-s ramp up and down. For the S-tDCS session, a 2 mA current was initially sent but ramped down to 0 mA over 30 s and maintained for the following 20 min, imitating the sensation of A-tDCS but not inducing any physiological response. Throughout all sessions, impedance was monitored and maintained below 10 kΩ to ensure consistent stimulation.

To assess the integrity of blinding, participants were asked after each session whether they believed they had received active or sham stimulation, and to report any sensations experienced (e.g., tingling, warmth, itching, or fatigue). No significant differences were reported in perceived discomfort or sensation intensity between the A-tDCS and S-tDCS sessions. The most common sensation across both conditions was mild tingling at the electrode site within the first 30 s of stimulation onset, which dissipated quickly. These responses suggested that the sham protocol was effective in maintaining participant blinding.

### 2.4 Golf swing performance assessment

All swing tests were carried out in a standard indoor golf room. A thick gray curtain was placed in the swing direction, with a red circle drawn at the center as a target. Performance metrics were recorded using a Doppler radar-based launch monitor (Trackman 4, Denmark), which captures the full swing and ball flight in 3D by measuring instantaneous clubhead and ball speeds, as well as computing derived metrics such as carry distance, face angle, and side deviation based on the launch angle, spin axis, ball velocity, and impact dynamics. Prior studies have examined the validity and reliability of the monitor in measuring swing performance metrics, with intraclass correlation coefficients greater than 0.87 (37, 38).

Trackman's carry distance is computed from measured launch data using a proprietary ball flight model that accounts for launch angle, spin rate, and atmospheric conditions. While the raw measurements (e.g., clubhead speed, ball speed) have high temporal fidelity (sampling rate = 40,000 samples per second), the derived values are estimates and may carry a larger margin of error, particularly in indoor settings. To minimize variability, all trials were conducted indoors under standardized lighting and temperature, with no wind, and consistent ball type and tee height across sessions (39). Trackman Performance Studio software (version: 7.0) were used for data acquisition.

Prior to each testing session, the Trackman was calibrated using the manufacturer's auto-level and alignment tools to ensure accurate horizontal and vertical tracking. Each session began with a short calibration warm-up (3–5 swings) to confirm radar-tracking consistency.

Each participant completed three swing tasks before and after each intervention session, whether A-tDCS or S-tDCS:

(1) Driver swing test: maximal driving distance,
(2) Iron 7 (34° loft): a balanced task requiring both long-driving distance capacity and accuracy,
(3) 100-yard wedge shot: optimal precision control.

The selection of tasks in this study was grounded in both golf swing mechanics and typical on-course tactical execution. The driver is conventionally used to maximize carry distance from the tee, representing the optimal long-driving distance capacity. The Iron 7 was selected as a dual-demand swing, requiring both moderate distance and directional accuracy, making it representative of mid-range approach shots. The 100-yard wedge shot was chosen to emphasize fine motor control, particularly distance regulation, trajectory shaping, and target precision. Although all three tasks cover distint carry distances ranging from 100 to 300 yards, they rely on full-swing mechanics with similar kinematic patterns. This consistency in swing structure across tasks was critical to ensure that observed differences in performance could be attributed to neuromodulatory effects of tDCS rather than confounding variations in swing type or effort level.

Each task consisted of 10 swing attempts, with a 60-second rest between each attempt. A certified strength and conditioning coach led the participants to warm up before the test, including a 5-minute dynamic stretch and 10 practice swings. During the test, a certified golf coach provided standardized oral instructions, such as “keep head steady”, to minimize technique variability and ensure the performance maximization.

The following swing performance variables were collected: (1) Long-driving distance capacity: clubhead speed, ball speed, and carry distance; (2) Accuracy-related: face angle, side distance. For wedge shot accuracy, the accuracy-related variables were calculated using side distance, carry distance, and target distance to get the horizontal, lateral, and radial error.

For data cleaning, the following procedure was applied: (1) trials with obvious signal dropouts, (2) miss-hits, or (3) data inconsistencies exceeding 3 SD from individual means were excluded. Fewer than 5% of trials were removed under these criteria. For consistency, performance variables were averaged from 3 valid swings selected from 5 consistent attempts (out of 10 total), excluding each participant's longest and shortest swings to avoid outliers and enhance within-subject reliability.

### 2.5 Statistical analysis

All data were processed using Excel and SPSS (Version 29.0, IBM). Outliers were checked using 3 median absolute deviation, and no data was excluded. To examine the effects of stimulation (A-tDCS vs. S-tDCS) and time (Pre vs. Post), we performed two-way repeated-measures ANOVAs for each dependent variable. Each ANOVA model included two within-subject factors: Condition (2 levels) and Time (2 levels). This approach allowed assessment of main effect as well as Condition × Time interactions.

When a significant interaction was observed, we conducted *post hoc* paired-sample *t*-test to evaluate within-condition difference (i.e., Pre vs. Post under A-tDCS and S-tDCS). Bonferroni correction was applied for multiple comparison (*α* = 0.05/2 = 0.025), and both raw and adjusted *p*-values are reported. For each *t*-test, we calculated Hedge's g as the effect size, along with the corresponding 95% confidence interval (CI) to improve interpretability, particularly given the small sample size (*n* = 8).

Assumption of normality were assessed via Shapiro–Wilk tests and visual inspection of Q-Q plots. Shapiro–Wilk tests revealed all variables were normally distributed (W ≥ 0.823, *p* ≥ 0.05), allowing the application of ANOVAs. Sphericity was not a concern due to the two-level design. Statistical significance was set as *p* < 0.05. Results are reported as mean ± standard deviation. To avoid overstating results, non-significant trends (*p* > 0.05) are reported cautiously and only interpreted when supported by robust effect sizes (Hedge's *g* > 0.8) and narrow CIs.

## 3 Results

This randomized, double-blind, crossover trial evaluated the acute effects of A-tDCS vs. S-tDCS on golf swing performance across three swing tasks: driver, iron, and wedge. Descriptive statistics for all variables are presented in Table 1. A series of 2 × 2 repeated-measures ANOVAs were performed for each variable, with Condition (A-tDCS vs. S-tDCS) and Time (Pre vs. Post) as within-subject factors. *Post hoc* analysis with paired *t*-tests was employed to further examine those variables with significant interaction. Detailed results of ANOVAs are reported below in the order per task.

**Table 1:** Descriptive summary of all variables.

Task	Variable	Condition × Time			
		A-tDCS-Pre	A-tDCS-Post	S-tDCS-Pre	S-tDCS-Post
Driver	Clubhead speed (mph)	100.93 ± 13.25	101.38 ± 12.9	99.4 ± 12.87	98.9 ± 12.48
	Face angle (°)	4.19 ± 2.82	3.33 ± 1.93	2.23 ± 1.5	2.44 ± 2.43
	Ball speed (mph)	148.45 ± 19.45	149.95 ± 19.85	148 ± 19.03	147.05 ± 18.88
	Side distance (yard)	16.31 ± 8.44	9.78 ± 3.83	12.95 ± 11.08	13.81 ± 8.7
	Carry distance (yard)	241.89 ± 38.37	246.66 ± 38.55	242.69 ± 35.61	241.2 ± 33.78
Iron	Clubhead speed (mph)	83.72 ± 10.27	84.51 ± 10.28	84.33 ± 8.92	83.12 ± 8.69
	Face angle (°)	2.5 ± 1.24	2.14 ± 1.12	2.66 ± 3.06	2.87 ± 1.89
	Ball speed (mph)	117.47 ± 14.56	119.31 ± 14.55	116.88 ± 12.67	115.71 ± 12.72
	Side distance (yard)	7.3 ± 3.43	4.02 ± 1.82	7.98 ± 3.98	7.82 ± 3.63
	Carry distance (yard)	163.96 ± 23.79	167.89 ± 24.67	162.09 ± 20.06	160.23 ± 20.49
Wedge	Vertical error (yard)	3 ± 2.98	6.23 ± 4.72	7.11 ± 7.17	10.36 ± 2.9
	Lateral error (yard)	3.94 ± 2.33	4.05 ± 1.78	3.77 ± 1.9	4.36 ± 1.9
	Radial error (yard)	5.31 ± 3.18	7.77 ± 4.42	8.99 ± 6.05	11.4 ± 2.8

Mean ± SD. A-tDCS, active transcranial direct current stimulation group; S-tDCS, sham transcranial direct current stimulation group.

### 3.1 Driver task

**Figure 3:**
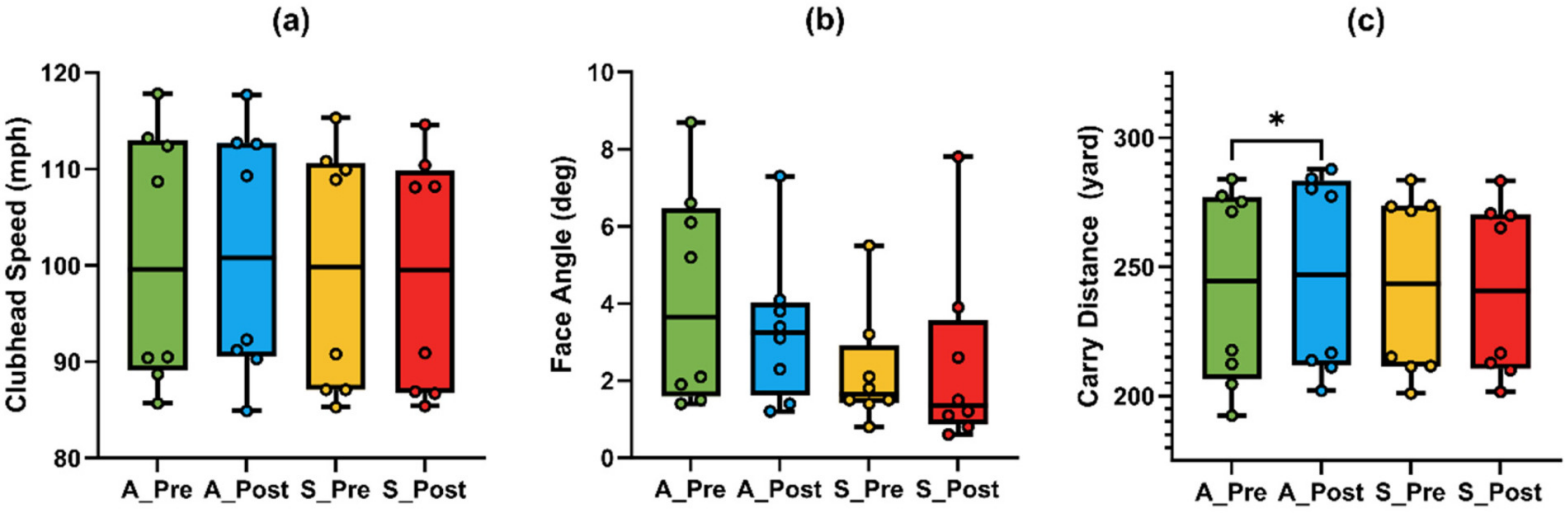
Comparison of golf swing performance metrics under anodal (A) and sham (S) tDCS conditions in the driver task. **(a)** Clubhead speed; **(b)** face angle; **(c)** carry distance. An asterisk (*) indicates a significant difference between pre- and post-tests under the A-tDCS condition.

Repeated-measures ANOVA (Table 2) revealed a significant Time × Condition interaction for clubhead speed [F _(1,7)_ = 9.757, *p* = 0.017, *η*^2^ = 0.582], with a significant main effect of Condition [F _(1,7)_ = 13.592, *p* = 0.008]. *Post hoc* test (Figure 3a) indicated a non-significant increase under A-tDCS [t_(7)_ = 1.34, *p* = 0.221, *g* = 0.42, 95% CI (−0.24, 1.06)] and a non-significant decrease under S-tDCS [t _(7)_ = –2.42, *p* = 0.046, adj. *p* = 0.092, *g* = –0.76, 95% CI (–1.47, −0.01)]. While the interaction was significant, within-condition changes did not reach corrected significance levels.

**Table 2:** Results of repeat-measures-ANOVAs for driver task.

Variable	Repeated-measures-ANOVA			
	Effect	F (1,7)	*p*-value	Partial *η*^2^
Clubhead speed (mph)	Condition	13.592	0.008*	0.66
	Time	0.011	0.918	0.002
	Interaction	9.757	0.017^#^	0.582
Face angle (°)	Condition	1.267	0.297	0.153
	Time	0.51	0.498	0.068
	Interaction	8.97	0.02^#^	0.562
Ball speed (mph)	Condition	2.971	0.128	0.298
	Time	0.892	0.376	0.113
	Interaction	4.993	0.061	0.416
Side distance (yard)	Condition	0.009	0.926	0.001
	Time	2.015	0.199	0.224
	Interaction	3.619	0.099	0.341
Carry distance (yard)	Condition	1.176	0.314	0.144
	Time	5.249	0.056	0.429
	Interaction	9.786	0.017^#^	0.583

* Significant main effect (*p* < 0.05).

^#^ Significant interaction effect (*p* < 0.05).

A similar pattern was observed for carry distance, which also showed a significant Time × Condition interaction [F _(1,7)_ = 9.786, *p* = 0.017, *η*^2^ = 0.583]. *Post hoc* tests (Figure 3c) revealed a significant increase in carry distance under A-tDCS [t _(7)_ = 3.66, *p* = 0.008, adj. *p* = 0.016, *g* = 1.15, 95% CI (0.28, 1.98)], while no change was observed under S-tDCS (*p* = .238, *g* = –0.41).

Face angle also showed a significant interaction effect [F _(1,7)_ = 8.97, *p* = .020, *η*^2^ = 0.562]. Although the *post hoc* comparisons (Figure 3b) did not reach significance after correction (A-tDCS: *p* = .194; S-tDCS: *p* = .555), the directional decrease under A-tDCS (mean change = –0.86°, *g* = −0.451) suggests potential improvement in accuracy that may warrant further exploration.

Other variables, including ball speed and side distance, did not show statistically significant interactions (*p* = .061 and *p* = .099, respectively), and *post hoc* comparisons yielded non-significant or small effects (Hedge's *g* < 0.70), and are thus not interpreted further.

### 3.2 Iron task

The ANOVA results for iron task are presented in Table 3. Repeated-measures ANOVA revealed a significant Time × Condition interaction for clubhead speed [F _(1,7)_ = 23.53, *p* = 0.002, *η*^2^ = 0.771], ball speed [F _(1,7)_ = 17.97, *p* = 0.004, *η*^2^ = 0.72], and carry distance [F _(1,7)_ = 18.53, *p* = 0.004, *η*^2^ = 0.726], indicating differential responses to A-tDCS and S-tDCS across time.

**Table 3:** Results of repeat-measures-ANOVAs for iron task.

Variable	Repeated-measures-ANOVA			
	Effect	F (1,7)	*p*-value	Partial η^2^
Clubhead speed (mph)	Condition	0.083	0.781	0.012
	Time	0.437	0.53	0.059
	Interaction	23.527	0.002**	0.771
Face angle (°)	Condition	0.221	0.652	0.031
	Time	0.033	0.861	0.005
	Interaction	0.801	0.401	0.103
Ball speed (mph)	Condition	0.679	0.437	0.088
	Time	1.34	0.285	0.161
	Interaction	17.971	0.004**	0.72
Side distance (yard)	Condition	1.828	0.218	0.207
	Time	7.678	0.028*	0.523
	Interaction	3.394	0.108	0.327
Carry distance (yard)	Condition	0.998	0.351	0.125
	Time	4.408	0.074	0.386
	Interaction	18.533	0.004**	0.726

* Significant main effect (*p* < 0.05).

** Significant interaction effect (*p* < 0.05).

**Figure 4:**
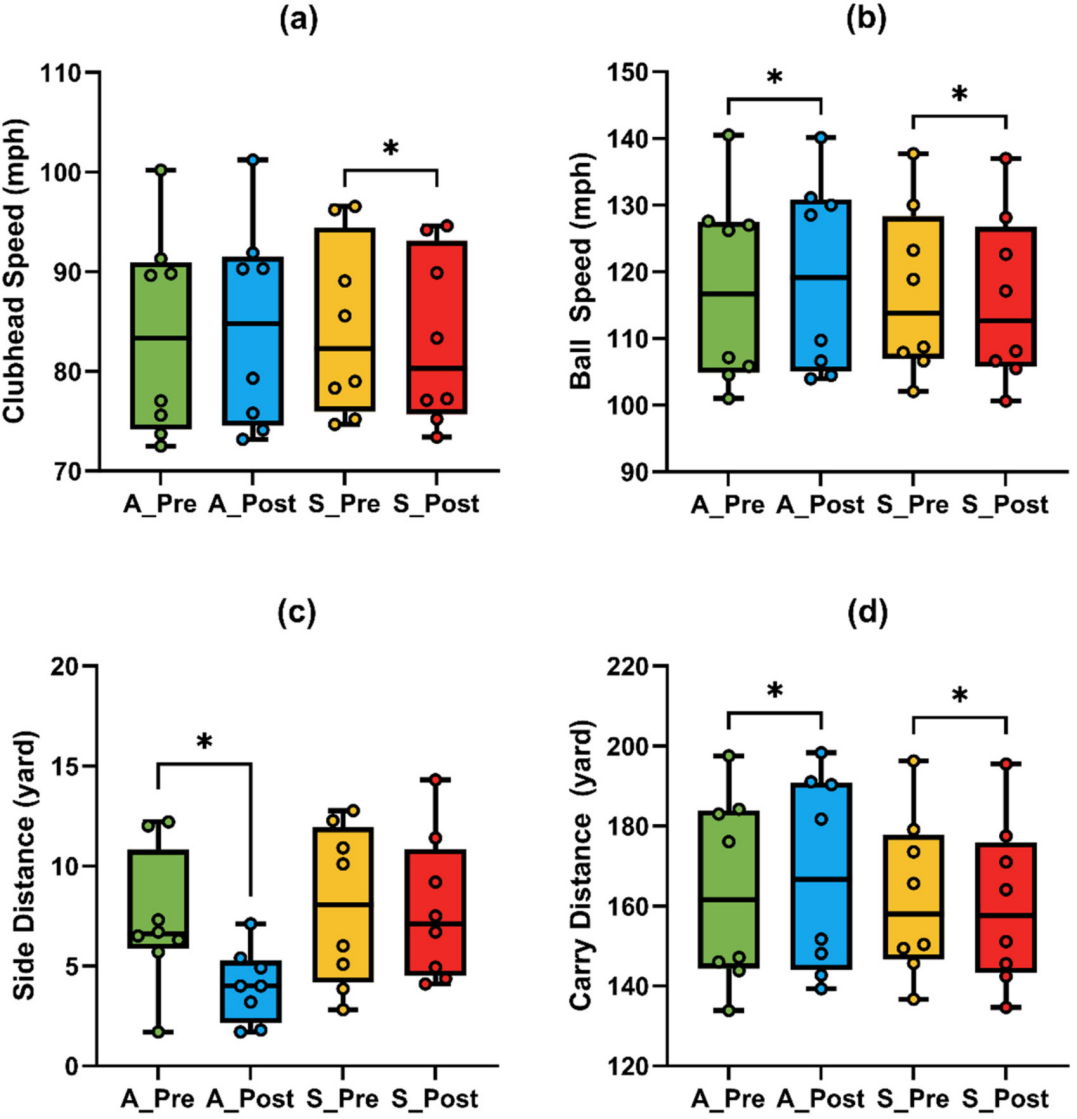
Comparison of golf swing performance metrics under anodal (A) and sham (S) tDCS conditions in the iron task. **(a)** Clubhead speed; **(b)** ball speed; **(c)** side distance; **(d)** carry distance. Asterisks (*) indicate significant changes between pre- and post-tests.

Post hoc comparisons revealed a significant reduction in clubhead speed (Figure 4a) following S-tDCS [t _(7)_ = –3.14, *p* = 0.016, adj. *p* = 0.032, *g* = –0.99, 95% CI (–1.76, −0.17)], while the increase under A-tDCS was not significant [t _(7)_ = 2.13, *p* = 0.071, adj. *p* = 0.142, *g* = 0.67].

Ball speed (Figure 4b) also showed significant pre-post changes in both directions: a significant increase under A-tDCS [t _(7)_ = 3.12, *p* = 0.017, adj. *p* = 0.034, *g* = 0.98, 95% CI (0.17, 1.75)] and a significant decrease under S-tDCS [t _(7)_ = –4.28, *p* = .004, adj. *p* = 0.008, *g* = –1.34, 95% CI (–2.25, −0.40)], indicating a robust stimulation effect on long-driving distance capacity.

Carry distance (Figure 4d) improved significantly following A-tDCS [t _(7)_ = 3.68, *p* = 0.008, adj. *p* = 0.016, *g* = 1.15, 95% CI (0.28, 1.99)] and decreased significantly under S-tDCS [t _(7)_ = –3.71, *p* = 0.008, adj. *p* = 0.016, *g* = –1.17, 95% CI (–2.00, −0.29)].

In terms of accuracy, side distance (Figure 4c) showed a significant Time × Condition interaction [F _(1,7)_ = 7.97, *p* = 0.028, *η*^2^ = 0.532]. A-tDCS led to a significant reduction in side deviation [t _(7)_ = –4.20, *p* = 0.004, adj. *p* = 0.008, *g* = –1.32, 95% CI (–2.21, −0.38)], while no change was observed under S-tDCS (*p* = 0.902, *g* = –0.04). No significant effects were found for face angle (all *p* > 0.35), and effect sizes were small (| *g* | < 0.5).

### 3.3 Wedge task

No significant Time × Condition interactions were observed for any of the accuracy variables in the wedge shot task (Table 4), including vertical error [F _(1,7)_ = 0.00, *p* = 0.995, *η*^2^ = 0.000], lateral error [F _(1,7)_ = 0.26, *p* = 0.628, *η*^2^ = 0.035], or radial error [F _(1,7)_ = 0.001, *p* = 0.975, *η*^2^ = 0.000]. Although a main effect of time was found for vertical error [F _(1,7)_ = 6.35, *p* = 0.040] and approached significance for radial error (*p* = .077), these effects did not differ between stimulation conditions and are therefore not interpreted further. No performance improvements were attributed specifically to A-tDCS or S-tDCS in this task.

**Table 4:** Results of repeat-measures-ANOVAs for wedge shot task.

Variable	Repeated-measures-ANOVA			
	Effect	F (1,7)	*p*-value	Partial η^2^
Vertical error (yard)	Condition	4.351	0.075	0.383
	Time	6.351	0.04	0.476
	Interaction	0	0.995	0
Lateral error (yard)	Condition	0.006	0.94	0.001
	Time	0.54	0.485	0.072
	Interaction	0.256	0.628	0.035
Radial error (yard)	Condition	3.9	0.089	0.358
	Time	4.299	0.077	0.38
	Interaction	0.001	0.975	0

## 4 Discussion

This study selected the golf swing as a model to examine whether tDCS targeting the M1 could acutely enhance golf swing performance across different task demands. Three representative swing tasks were selected as the model for their distinct neural patterns, including maximal distance demanded driver swing, moderate distance- and precision-required iron swing, and optimal accuracy-depended wedge shot (40). Importantly, all three swing types employed full-swing mechanics, ensuring that performance changes could be attributed to neuromodulatory effects rather than differences in motor pattern or task structure.

Using a double-blind, crossover design and 2 × 2 repeated-measures ANOVA, the results revealed that M1-targeted anodal A-tDCS elicited acute and significant improvements in long-driving distance capacity: ball speed and carry distance in iron tasks and carry distance in driver task. The robust improvements were accompanied by large effect sizes (Hedge's *g* > 0.8), reinforcing the role of M1 stimulation in boosting gross motor output (24). The observed improvements in driver and iron tasks align with previous studies reporting A-tDCS over M1 area elevates cortical excitability, therefore improving performance by facilitating force production and technique execution in tasks requiring rapid movement execution (20, 41). Moreover, the strong effect sizes in long-driving distance capacity post A-tDCS support that neuromodulation may facilitate explosive strength in real-world sporting contexts, which is especially important in golf, where even minor gains in distance can tremendously impact the ultimate competitive scores (10).

Furthermore, ball speed and clubhead speed significantly declined under the sham condition in the iron task, while remaining stable or improving under A-tDCS. These opposing trends served as complementary findings, strengthening the conclusion that A-tDCS enhances force generation and may also counteract performance fatigue during repetitive tasks (24). Performance drops under sham may reflect session-to-session variability or mental fatigue rather than any physiological effect of the sham protocol itself. Such effects are particularly relevant in sport settings where neuromuscular efficiency and fatigue resistance are crucial (6, 42).

Interestingly, A-tDCS also led to a significant reduction in side deviation in the iron task, suggesting potential accuracy gains. However, traditional knowledge about M1 is its association with motor execution instead of fine motor control or visuospatial processing (43, 44). We propose M1 stimulation optimizes efferent drive timing, reducing kinematic variability during downswing transfer (27). This may partly explain the concurrent improvements in both long-driving distance capacity and directional control observed in iron task. Moreover, these findings challenge the traditional view that force production and accuracy rely on entirely separate neural mechanisms (45, 46). However, face angle changes, though trending favorably in the driver task, did not reach statistical significance, and are interpreted cautiously.

In contrast, M1-targeted tDCS was limited for tasks that heavily rely on cerebellar-parietal networks, as evidenced by the lack of significant stimulation-specific effects in the wedge task, where high spatial accuracy is dominated over force production. Identical improvements in both conditions confirm practice effects dominated over neuromodulation in this precision task. This finding aligns with the traditional dichotomy that matching stimulation targets to task-specific neural demands is important to address distinct issues (47). Tasks like wedge shots rely on precise sensorimotor integration and visuomotor coordination (48).

The decision to target M1 in this study was based on its well-established role in modulating corticospinal excitability and voluntary motor output, particularly in power-dominant tasks (23, 24). However, the absence of stimulation-specific effects in the wedge task highlights the limitations of this approach for precision-dominant movements. Tasks requiring fine motor control and spatial accuracy may depend more on cerebellar and parietal regions, such as the posterior parietal cortex, which are involved in sensorimotor integration and visuomotor coordination (49). While M1 was selected for its practicality and known safety in sport stimulation protocol (50), future studies should consider dual-site or network-based approaches, targeting both M1 and posterior parietal cortex or cerebellum, to address the full complexity of goal-directed movements in sport (51).

The results found in this study provide several practical implications for applying tDCS in golf training. For professional golfers, the task-specific gains in long-driving distance capacity make M1-tDCS a possible tool for warm-ups before competitions, and a potential supplementary approach to maximize long-driving distance capacity with minimal physical loads. For recreational players, tDCS may offer a safer path for practicing and performance improvement, thus reducing the cumulative mechanical stress from repetitions. This is crucial to maintain their health condition and ensure the lifelong engagement in physical activities, especially under the background that golf globally attracts more than 60 million population (1).

Nevertheless, several limitations must be acknowledged. Firstly, though a satisfactory trend and significance was observed, the sample size is small and the participants is professional golfers, which restricts the generalizability of this study. However, this homogeneous sample was chosen to minimize interindividual variability, allow for accurate detection of acute stimulation effects, and provide the empirical evidence for future insightful research. Secondly, only acute effects were investigated in this study. It is still unknown whether long-term A-tDCS could induce better enhancements or it may face ceiling effects, and whether regular application of A-tDCS could reduce the training volume. Thirdly, the exclusive stimulation of M1 limits the interpretation of the neural mechanisms involved in accuracy improvements, especially in the absence of neuroimaging or neurophysiological data, such as EEG or TMS. Lastly, the potential placebo effect, although minimized through a double-blind, sham-controlled design, cannot be entirely ruled out. Further research may explore the chronic effects of repeated tDCS, as well as dual-site or high-definition tDCS, on larger sample sizes and broader golf populations, such as novice and master players. Moreover, biomechanical and neuromuscular measurements could be incorporated in future studies to explore the potential mechanisms underlying the observed performance enhancements in this study.

## 5 Conclusion

In conclusion, this study provides evidence that M1-targeted anodal tDCS can acutely enhance performance in golf swing tasks requiring explosive motor output, with some potential benefit to directional accuracy in tasks that integrate both power and precision. However, limited improvements were found in tasks highly dependent on precision control, which highlights the importance of intervention strategies in neural-based performance studies. These findings suggest that tDCS may serve as a valuable tool in sport training, especially for those demanding maximal and rapid force generation. By bridging the gap between motor performance requirement and injury prevention, tDCS may contribute to safer, more efficient training paradigms in both elite and broader athletic populations.

## Data Availability

The raw data supporting the conclusions of this article will be made available by the authors, without undue reservation.
